# Environmental Harmony and Evaluation of Advertisement Billboards with Digital Photogrammetry Technique and GIS Capabilities: A Case Study in the City of Ankara

**DOI:** 10.3390/s8053271

**Published:** 2008-05-19

**Authors:** Cevdet C. Aydın, Recep Nişancı

**Affiliations:** 1 Hacettepe University, Büklüm Sokak 74/10 Küçükesat / Ankara, Turkey; E-mail: ceaydin@hacettepe.edu.tr; 2 Karadeniz Technical University, Department of Geodesy and Photogrammetry; E-mail: rnisanci@ktu.edu.tr

**Keywords:** Urban beauty, environmental harmony, advertisement, visual pollution, digital photogrammetry, GIS

## Abstract

Geographical Information Systems (GIS) have been gaining a growing interest in Turkey. Many local governments and public agencies have been struggling to set up such systems to serve the needs and meet public requirements. Urban life shelters the advertisement reality which is presented at various places, on vehicles, shops etc. in daily life. It can be said that advertisement is a part of daily life in urban area, especially in city centers. In addition, one of the main sources of revenue for municipalities comes from advertising and notices. The advertising sector provides a great level of income today. Therefore advertising is individually very important for local governments and urban management. Although it is valuable for local governments, it is also very important for urban management to place these advertisement signs and billboards in an orderly fashion which is pleasing to the eye. Another point related to this subject is the systematic control mechanism which is necessary for collecting taxes regularly and updating. In this paper, first practical meaning of notice and advertisement subject, problem definition and objectives are described and then legal support and daily practice are revised. Current practice and problems are mentioned. Possibilities of measuring and obtaining necessary information by using digital images and transferring them to spatial databases are studied. By this study, a modern approach was developed for urban management and municipalities by using information technology which is an alternative to current application. Criteria which provide environmental harmony such as urban beauty, colour, compatibility and safety were also evaluated. It was finally concluded that measuring commercial signs and keeping environmental harmony under control for urban beauty can be provided by Digital Photogrammetry (DP) technique and GIS capabilities which were studied with pilot applications in the city center of Ankara.

## 1. Introduction

Planning and keeping alive modern and healthy urbans can only be possible with the making of serious designing in different expertise fields and establishing serious control mechanism for municipalities and local governments. One of these expertise fields is notice and advertisement subject which is taxable and has to be seriously considered in many factors such as; urban beauty, visual harmony and design compatibility for urban managements.

Notice and advertisement subject has a commercial meaning for urban life. Today billions of dollars expenses have been made for advertisement by private and government organizations. In addition, rivalry and advertisement investment are increasingly growing up in Turkey [21]. It is an important subject for tax revenue and income source and on the other hand it has to be under controlled in order not to cause to visual pollution. Also updating stage of sign and advertisement information is necessary to collect taxes safely.

Photogrammetry is the process of obtaining quantitative two and three-dimensional information about the geometry of an object or surface through the use of photographs. The DP served several digital products to GIS in vector, raster and surface form such as digital map, ortho-images and digital elevation model [11]. Digital close range photogrammetry (DCRP) accurately measures objects directly from photographs or digital images captured with a camera at close range [3-9]. Using these products of DP and image processing techniques 3D models can be generated and transferred into the GIS.

Legal arrangements were made about signs ordinance and advertisement structures in 1981 in Turkey. In this ordinance, details of the standards related to collecting signs taxes were explained. According to the standards “any kind of signs including advertising structures commonly known as billboards, signs, signboards, canopies, marquees, neon, electric and illuminated signs, or any other advertising devices of any kind are taxable materials in municipality borders”. Also variety and amount of taxes, fees and exceptions are explained [12].

In Turkey, especially municipalities have serious problems in obtaining necessary geometric and attribute sign information and manage them for tax income and environmental harmony. In big metropolitans, there are billions of taxable signs, billboards etc which cause to visual pollution and tax evasion. Also a lot of serious accidents have come to exist due to not fixing billboards on building facades in a proper way. To overcome all these problems, sign owners declarations must be inspected by local governments or municipalities not only sign measurement for taxes but also other criteria such as; environmental harmony, visual pollution, safety risks and design compatibility. DCRP can solve these problems, because it provides reliable technique of metric measurement in a fast, precise and inexpensive way and GIS provides data collection, data management and necessary queries without consuming much time.

In this study, municipalities and local governments in different part of world sign ordinances, sign permit applications and regulations are examined and evaluated. When their sign ordinances are examined, it is clearly seen that all municipalities have their own similar laws and update them with the new developments. They have also esthetic departments to determine the application suitability. Building inspector mechanism works properly by going to place where sign is located. Although visual criteria are very important for the municipalities, they have applied traditional approach for valued commercial sign of the building. However outside morphology of building has not been quantitatively evaluated and there is no specific analytical evaluation for metric quality. Therefore we have offered a relatively simple and applicable approach for the geometric evaluation of the commercial sign to be mounted on the sights of building. Also a similar study was performed for Turkey's condition and sign ordinance and some researches [16-18] are considered.

The objective of this paper is to develop a new approach for dynamic control mechanism and evaluation method to obtain metric measurements for notice and advertisement billboards which are physically non approachable including DP and GIS capabilities. The importance of data collection, the kinds of data to be collected by using high resolution digital camera images, the method of measuring, evaluating and revaluing signs sizes using DCRP technique with 2D and 3D digital photogrammetric maps and the attributes to be presented in the database were all studied. Pilot applications and examples were all examined in the case study area situated in the capital city of Turkey.

## 2. Environmental Harmony Criteria and Definitions

Local managements have serious control steps for visual criteria and tax revenue in abroad. Especially city beauty and harmony is one of the vital subject as well as tax and etc. In Turkey, Municipalities have become conscious about the factors in recent years. In the following stage, some definitions and criteria such as measurement of sign, design compatibility, colour, safety risks and materials will be explained related to the subject.

### 2.1. Measurement of sign area

**Figure 1.**
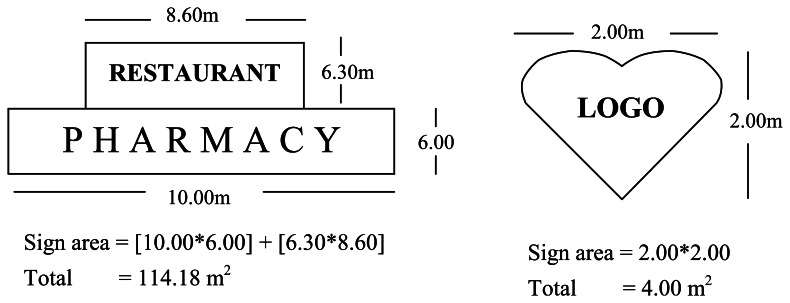
Sign area measurement.

The surface area of a sign shall be calculated by enclosing the extreme limits of all lettering, background, emblem, logo, representation or other display within a single continuous perimeter composed of squares or rectangles with no more than eight lines drawn at right angles. According to legal arrangement mentioned above, some sign measurement examples in different shape and scale shown in Figure 1. Figure 1 explains where a sign consists of one or more three-dimensional objects (i.e., balls, cubes, clusters of objects, sculpture, or statue-like trademarks), the sign area shall be measured as their maximum projection upon a vertical plane.

### 2.2. Design compatibility

Signs should make a positive contribution to the general appearance of the street and commercial area in which they are located. A well-designed sign can be a major asset to a building and can help urban beauty. Municipalities encourage imaginative and innovative sign design to develop their own sign application procedure for creating more orderly appearance and consistent size.

**Figure 2.**
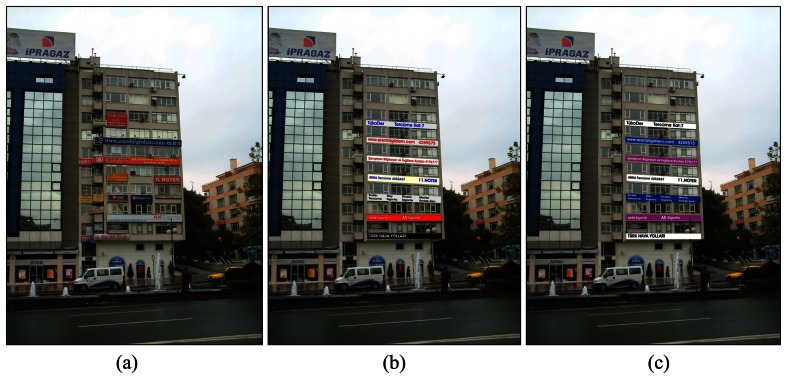
Sign placement and design compatibility; (a) is the original photo of the building facade taken from Kızılay square in Ankara and caused a chaotic appearance, (b) and (c) shows two different alternatives after design compatibility.

The scale of signs should be appropriate for the building. Size and shape of a sign should be proportionate with the scale of the structure. Signs should not obscure architectural features. Their design should be integrated with the design of the building. A well-designed building facade is created by the careful coordination of sign and architectural design and over-all colour scheme. Signs should also be cleaned, well-kept and painted. All signs should be checked by owners at least once in a month to keep them safe, clean, free of visible defects and functioning properly at all times (Figure 2).

**Figure 3.**
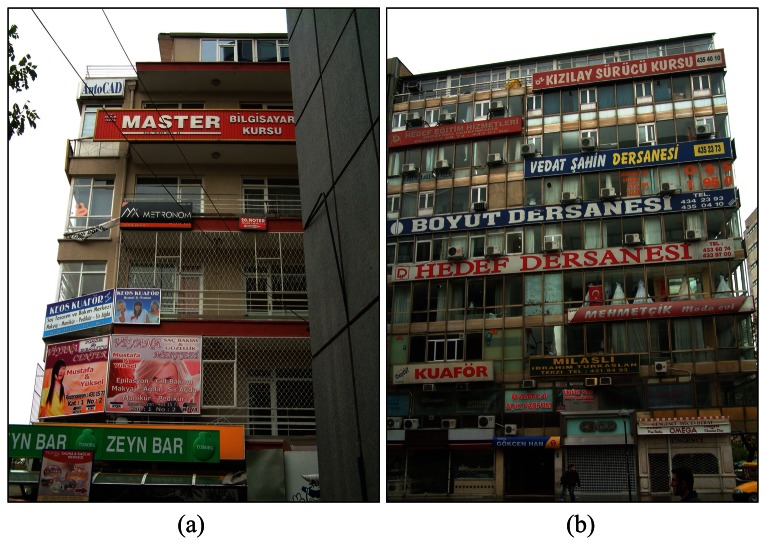
Visual pollution on building facades, (a) shows different scale and materials of signs with irrelevant colours, (b) presents a visual clutter on building facade.

In Turkey, Municipalities have started to struggle with visual pollution [22-23]. There are some building facades which have no appropriate design rhythm, no colour legibility, harmony and no scale or proportion (Figure 3).

There are no criteria for this building's facade and no inspections. These photos were taken from the most common place of the city centre in Ankara. Because of the lack of staff, it is difficult to control all buildings facades. These facades should be determined and designed in orderly appearance, kept clean and painted. In fact, it is not difficult to design all this facades with digital camera images and all necessary geometric and attribute information can be collected, evaluated with DP and image processing techniques. In application stage a small model was generated and transferred into the GIS with this approach.

### 2.3. Colour

Colour is one of the most important aspects of visual communication. Also it provides visual and environmental harmony, which is a very important factor for urban beauty. It can be used to catch the eye or to communicate ideas or feelings. So colours should be selected to contribute to legibility and design integrity. Poor colour selection causes poor communication. On the contrary, too many colours used thoughtlessly can confuse and negate the message of a sign.

**Figure 4.**
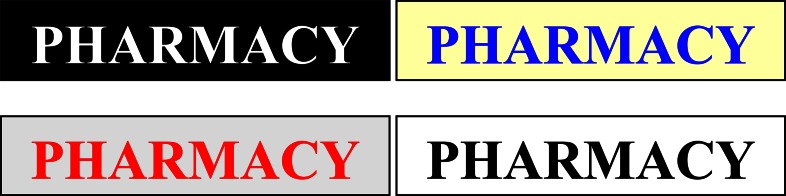
Contrasting letters of colour and background.

Contrast is an important influence on the legibility of signs. A substantial contrast should be provided between the colour and material of the background and the letters or symbols to make the sign easier to read in both day and night. Light letters on a dark background or dark letters on a light background are most legible (Figure 4).

### 2.4. Safety risks and materials

Sign materials can be wood (carved, sandblasted, etched, and properly sealed, primed and painted, or stained), metal (formed, etched, cast, engraved, and properly primed and painted or factory-coated to protect against corrosion), high density pre-formed foam or similar material. The very important point is that sign materials should be compatible with the design of the face of the facade and signs should be mounted safely where they placed. Especially in windy weather some sign accidents might be possible. Signs can fall down over pedestrians and customers.

One example to these problems took place on Saturday, January 01, 2007 in Mersin Province [24]. A huge billboard fell down because of wind and two teenage students were injured one of them being serious. Another one occurred in Istiklal Street in Istanbul. An elderly pedestrian was seriously wounded by a metal billboard on 24 March 2007 [25]. Many such incidents may be found in newspapers in Turkey. So the criteria such as safety risk are very important and must be kept under controlled. Safety risk can be scaled 1 to 10 and inspectors can evaluate signs safety criteria and enter values in GIS databases. In case of an emergency or a dangerous situation inspectors must send a team to sort the problem out to avoid accidents.

## 3. Evaluating Process of the Signs by Digital Photogrammetry

The successful development over recent years of DCRP is being started when it is applied in many fields. One of this application fields is to obtain metric information from object surfaces using high resolution digital images. Especially taxable commercial sign, advertisement notice boards etc. can be measured with DP technique using high resolution digital cameras and necessary metric information obtained rapidly and safely then stored in GIS databases [1].

### 3.1. Data acquisition with digital cameras

Digital camera images have relatively a number of possible advantages in creating and managing metric information.

- Digital images and GIS are crucial in the commercial and residential real estate market. Many realtors have discovered the value of showing available residential and real estate property on a map to display property digital and video images along with the map. It is also possible to access real estate information portals via the internet which presents digital and video images [7-19].
- Digital images of historical, tourist and cultural areas or buildings are very useful when used in digital map based GIS applications [9-13].
- Digital images can be used in metric applications. The digital images of object surfaces can be taken and evaluated. Then all the necessary metric information can be extracted from the photographs and stored in a database. Roof overhang measurements, width and length measurements of commercial posters and advertisement billboards, any object with surface measurements etc. can be obtained using DCRP with digital images.
- Photorealistic textures are used by covering 3D objects while forming 3D models in a computer environment. Texture information is taken directly from the digital camera images [5-10].
- Digital images can be used especially in the presentation of buildings not suitable for ordinary map preparation. In the last decade, with developing architecture and construction techniques very complex buildings have been constructed. It is very difficult to present these buildings on digital maps. Therefore these kinds of complicated buildings can be presented in GIS applications using digital camera images.
- Digital images are used effectively in the reconstruction of historical monuments.

Digital cameras are ubiquitous, have ever higher resolutions and are quite suitable for accurate measurement required in architectural and archaeological recording, forensic measurement and engineering documentation and in numerous other application domains. In application stage, a calibrated 5.7 mega pixel digital camera was used in this study. Photogrammetric evaluation of the images and camera calibration were performed with PhotoModeler Pro 4.0 DCRP software.

### 3.2. Photogrammetric Processes

For many years photogrammetric procedures have significantly contributed to the touchless measurement of objects of all kind. Especially the introduction of digital image processing techniques has opened new fields of applications for photogrammetry. Digital image processing is an efficient tool to derive geometrical information from digital imagery in a fast and economic way. In recent years versified approaches for object recognition as well as ways for extraction of geometrical information were developed for very different applications [15].

An important feature of the bundle adjustment algorithm used in photogrammetry is that it will automatically determine the locations and orientations of the cameras used to make the photogrammetric measurements. Once the photographs have been taken, they are loaded into the PhotoModeler software package. Within the software, the camera calibration parameters are identified and points of interest are marked on the photographs. Corresponding points on different images are then referenced to each other. Referencing tells the software that point A in picture 1 is the same physical point as point B in picture 2. When a minimum number of points have been marked and referenced (approximately 8-10 points per photo). The 3D coordinates of the referenced points as well as the camera locations and orientations are calculated by using the bundle adjustment. Additional points can be marked and referenced and the bundle adjustment re-executed until all points of interest have been measured. This was an iterative process with the positions and angles of the camera stations and the positions of the marked points being adjusted after each iteration, until a satisfactory solution was achieved. After this stage data can be exported and studied with other software packages.

The accuracy of a final measurement in PhotoModeler depends on a number of factors: the resolution and number of photos, the angles between the photos, the number of referenced points, and the quality of the camera description. Initially only three photographs were used to produce the 3D model. Additional photographs were added later to improve accuracy and increase details [4-8-14-17].

## 4. Case Study

**Figure 5.**
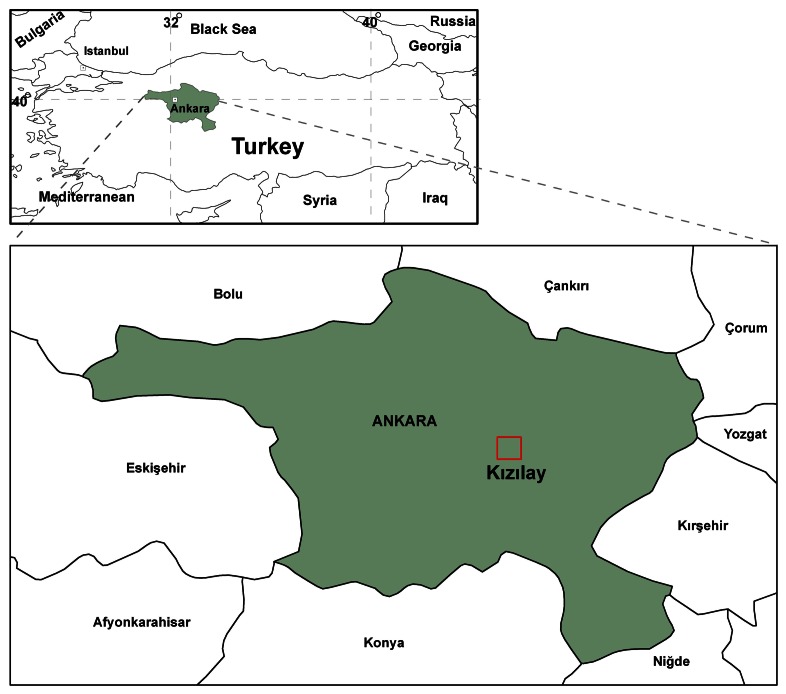
Location of the study area.

In order to apply and evaluate the selected approach, the following study was carried out in Tunalı Hilmi Street in Ankara Province (Figure 5). During the application stage, 2D and 3D digital photogrammetric maps were obtained, application area was determined, high resolution digital camera images of signs and advertisement billboards were taken and transferred into the database, images were evaluated with DCRP software called PhotoModeler and all necessary measurements were performed. Eventually small GIS applications fulfilled by using geometric and attribute data.

**Figure 6.**
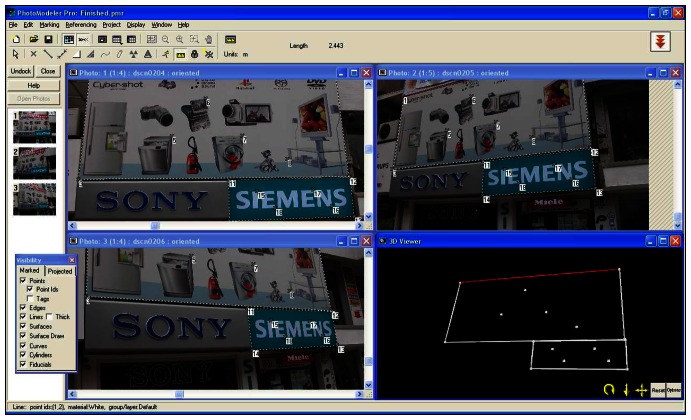
Camera shooting with minimum of tree photos.

The images were acquired by means of Nikon coolpix 5700-5.7 mega pixel digital camera which had already been calibrated for previous works. The photos should be taken considering the following points: each element depicted must be contained in a minimum of three photos (Figure 6.). The convergence between photos taken from different positions will have to have optimum values of 90° (good values of 60°) so that the beam adjustments are carried out well; there must be at least 50% overlap between photos.

Figure 6 represent points marked for each sign, processed and 3D model of signs generated. The software was used for the digital orientation and measurement of photographs. The mathematic model of relation between image coordinates and ground coordinates is the collinearity condition. Orientation parameters and ground point coordinates are obtained by means of this condition. The processing stage was performed with interior orientation, exterior orientation, relative orientation and absolute orientation respectively. The resulting models containing metric information and the 3D models are available to be exported in conventional formats (dxf, vrml, etc.) into other programs in order to be visualized, edited or processed. Another feature of the system is its capability to generate 3D models of surfaces and the subsequent projection of real textures, captured from the object's photographs, onto those models of surfaces [6].

During the application stage, street based building photos were taken and transferred into database. After processing, geometric and attribute data indicated on declaration form (square measures of signs, type of signs like animated sign, free standing sign, illuminated sign etc., address and commercial communication information etc.) obtained (Table 1) from photos and directly stored into GIS database. Table 1 contains necessary information from local inspection forms. These information are changeable depend upon municipalities legal arrangements.

**Figure 7.**
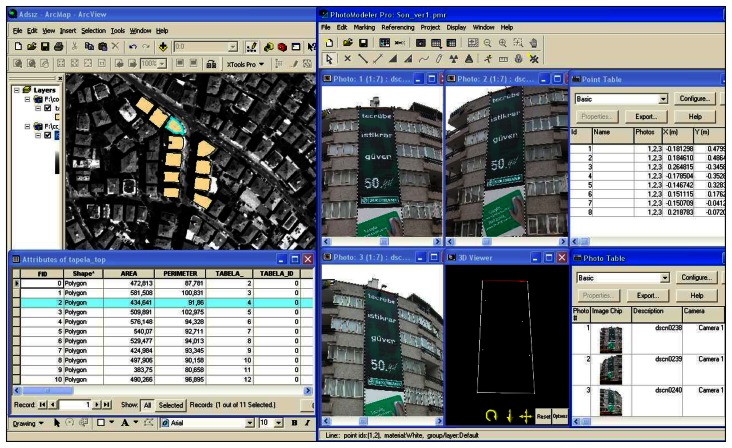
Metric and attribute information in databases.

Buildings facade and sign images are related with geometric information through GIS therefore all operations performed in a very short time and quickly/safely. Also metric information was stored in GIS database by using ArcGis 9.1 GIS software. Also 1/5000 scaled ortophoto images, 3D digital photogrammetric maps were used for evaluation stage and all billboard measurements and details were taken from photos (Figure 7).

**Figure 8.**
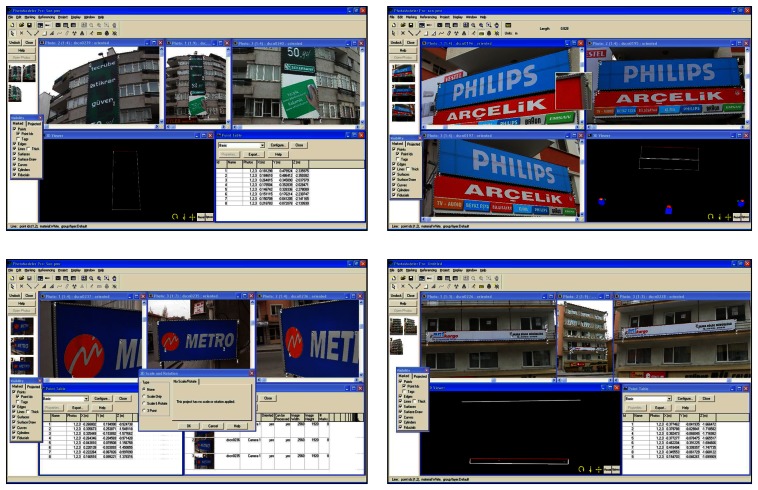
Whole process.

It is possible to make any measurement using images on condition that at least one measure value of the object or any point of xyz coordinates must be known to scale the image. In practice, this value can be taken from 3D digital photogrammetric maps used as base maps in GIS applications. All measures of accuracy controls and comparisons of original and measured values were performed and cm level of accuracy was obtained and the values found to be significant and acceptable (Figure 8).

Figure 8 shows some sign measurements of study area. Whole process was performed with 35 trio of photos (105 photos). Process stages lasted just a couple of day. The relative orientation was made at least 8 points for each photo and 804 point used in average for all photos. Some facades have more than one signs therefore some additional points were used for per photo.

**Figure 9.**
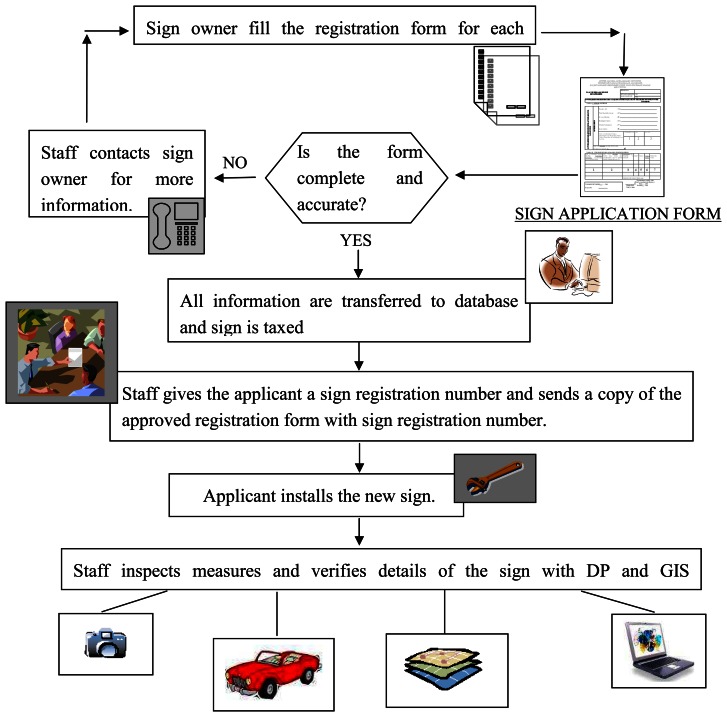
Workflow for tax collection and visual criteria.

Municipalities have not adequate experienced staff. Therefore legal inspection stage is getting difficult in practice. What we offer is an approach that mobile teams which consist of skilled staff can take all the signs photographs with high resolution digital cameras in municipality borders and then can evaluate them by means of DP and GIS capabilities. That provides a serious control mechanism, economic and easy work with less staff than usual. Figure 9 indicates the workflow about whole process.

All geometric and attribute information are related with address and numerating data. Therefore a spatial based address information system is a very vital component for practice applications [20- 2].

## 5. Conclusions

Tax revenue is one of the vital components and income for municipalities. In this study, an evaluation approach has developed especially for municipalities and local governments to collect tax revenue information regularly using high resolution digital camera images. The combination of DP and digital camera images provided a coupling opportunity for collecting data necessary for sign and advertising objects to be represented in a GIS environment. During the applications, cm level of accuracy was obtained between the original measurements and the measurements taken from photos. Also we were able to collect the data rapidly, safely and then to store it directly in GIS databases.

Using this approach, municipalities can take over of solving tax collection of signs and prevent tax evasion. Every queries, updates and necessary inspections can be handled with DP and GIS advantages. Also updating stage is very easy when photos are used. Photos taken date indicate last updating date of sign inspection.

At the end of the study, some serious deficiencies have determined in Turkey's conditions.

- During the studies it has realized that “sign ordinance” subject is being considered importantly by local governments and municipalities in all over the world to the contrary Turkey's situation. Especially environmental harmony criteria, collecting taxes etc. stages are seriously evaluated and any details taken into consideration. It has concluded that municipality's authority should make serious chances on related law and current applications.
- There are some social end economical differences between districts in municipality's border. These differences directly affect the commercial revenue. So, tax values should be revised with these differences and advertisement zones should be established and each zone must be evaluated individually.
- In Turkey and most of the world countries taxes are collected with declaration and municipality's authority carry on inspection stage. In turkey, sign taxes are collected once in a year. One of the most important difficulties in collecting stage is determining the unregistered advertisements. According to the law, taxable advertisements must be inspected. During the studies some interviews were made municipality's authority about the subject and it is concluded that the inspection stage can not be carried on orderly, healthy and systematically because of not having qualified and sufficient stuff. Considering all these reasons, mobile inspection teams can be established and a systematic control mechanism should be carried on by the teams during year.
- Because of the lack of stuff, inspection stage can be done by private sector for municipalities and local governments. This can provide healthy control mechanism.
- Each advertisement subject must have a unique registration number and related to its image and address/numerating information. Therefore all taxable advertisements inventory can be listed in database. This process provides great comfort for collecting taxes. Inspections teams should have mobile computers for using the evaluation method and shelter all advertisements registration numbers, images, measurements, last update date's and criteria stored in spatial GIS database. So when the mobile teams meet a new advertisement element they can check its registration number in a very short time and safely.
- During the study, it has determined that some important and necessary information about relevant laws, paying sign and advertisement tax, paying conditionals, process, periods and etc are needed. Solving all these problems out, information brochures should be prepared and necessary information, wide explanations and forms should be presented in municipality's official web sites.
- As mentioned in legal context stage above visual criteria are a very important component of visual communications. Decreasing and controlling of visual pollution some standards can be formed especially definite occupation groups such as doctors, pharmacies, lawyers etc.

It is obvious that the data collecting stage is very difficult and tedious work. On the other hand, the collected data must be reliable and straightforward. During the applications, it was observed that all the necessary geometric and attribute data relevant commercial advertisements were collected easily from digital camera images. So, lots of tedious work involved in data collecting stage can be diminished easily.

It has finally concluded that the evaluation method with DP, 2D-3D digital photogrammetric maps, high resolution digital camera images and GIS capabilities can be facilitated/diminished municipalities sign and advertisement revenue department's tedious work. All sign measurements and criteria can easily be collected and evaluated.

## Figures and Tables

**Table 1.** Sign and advertisement information.

**Sign**	**Geometric and Attribute Data**
	
Issued by	Date
Address information	Avenue, street, building number, door number
Sign type	Awning/canopy sign, blade/bracket sign, free standing sign, billboard, portable sign, illuminated sign, temporary sign, animated sign, building/wall sign, monument sign, banner
Description of sign (metric information)	Registration ID, sign height, sign area size (width- length), total sign area, number of sign faces, sign setback, sign material, sign colour, colour compatibility, sign safety, sign design capability, sign appearance, sign cleanliness
Sign owner information	Full name, phone, fax, e-mail, address, gsm
Description of property	Address of sign, property type, property zone
Type of premises	Commercial, professional or business office, retail/wholesale business, industrial, agriculture, gasoline or service station
Sign information	Name, address, telephone, fax, e-mail, sign image, sign video sequences
Message to be displayed	Highland Park – Orkney Islands
Inspection stage	Inspector name, date, number, time
Warnings	First, date, time, second, date, time
Other signs on property	Can be present by property images
Zoning	Zone 1,2,3
Penalties	Date, time
